# Improving Soluble Phenolic Profile and Antioxidant Activity of Grape Pomace Seeds through Fungal Solid-State Fermentation

**DOI:** 10.3390/foods13081158

**Published:** 2024-04-11

**Authors:** Yuzhu Zhao, Doudou Liu, Jiaxuan Zhang, Jiaxin Shen, Jiamin Cao, Huawei Gu, Mengqing Cui, Ling He, Gong Chen, Shuwen Liu, Kan Shi

**Affiliations:** 1 College of Enology, College of Life Sciences, College of Horticulture, Shaanxi Engineering Research Center for Viti-Viniculture, Viti-Viniculture Engineering Technology Center of State Forestry and Grassland Administration, Heyang Experimental and Demonstrational Stations for Grape, Ningxia Helan Mountain’s East Foothill Wine Experiment and Demonstration Station, Life Science Research Core Services, Northwest A&F University, Xianyang 712100, China; juicy@nwafu.edu.cn (Y.Z.); liudoudou@nwafu.edu.cn (D.L.); zhangjiaxuan@nwafu.edu.cn (J.Z.); 18735511021@163.com (J.S.); caojiamin@nwafu.edu.cn (J.C.); guhuawei@nwafu.edu.cn (H.G.); 19502910335@163.com (M.C.); heliurui@nwsuaf.edu.cn (L.H.); liushuwen@nwafu.edu.cn (S.L.); 2 School of Environmental Ecology and Biological Engineering, Wuhan Institute of Technology, Wuhan 430205, China; chengong@wit.edu.cn

**Keywords:** grape pomace seeds, valorization, phenolics, solid-state fermentation, *Monascus anka*, bioactivities

## Abstract

Grape pomace seeds contain abundant phenolic compounds, which are also present in both soluble and insoluble forms, similar to many other plant matrices. To further increase the extractable soluble phenolics and their antioxidant activities, grape pomace seeds were fermented with different fungi. Results showed that solid-state fermentation (SSF) with *Aspergillus niger*, *Monascus anka*, and *Eurotium cristatum* at 28 °C and 65% humidity had a significantly positive impact on the release of soluble phenolics in grape pomace seeds. Specifically, SSF with *M. anka* increased the soluble phenolic contents by 6.42 times (calculated as total phenolic content) and 6.68 times (calculated as total flavonoid content), leading to an overall improvement of antioxidant activities, including DPPH (increased by 2.14 times) and ABTS (increased by 3.64 times) radical scavenging activity. Furthermore, substantial changes were observed in the composition and content of individual phenolic compounds in the soluble fraction, with significantly heightened levels of specific phenolics such as chlorogenic acid, syringic acid, ferulic acid, epicatechin gallate, and resveratrol. Notably, during *M. anka* SSF, positive correlations were identified between the soluble phenolic content and hydrolase activities. In particular, there is a strong positive correlation between glycosidase and soluble phenols (r = 0.900). The findings present an effective strategy for improving the soluble phenolic profiles and bioactivities of grape pomace seeds through fungal SSF, thereby facilitating the valorization of winemaking by-products.

## 1. Introduction

Grape pomaces, accounting for 20–30% of the weight of grape berries, are winemaking by-products produced in massive quantities on a global scale. Grape pomace seeds make up approximately 38% to 52% of the weight of grape pomaces. Due to the abundance of bioactive compounds in grape pomace seeds, particularly phenolic compounds, which comprise 5% to 8% of their weight, there has been widespread interest in exploring their potential applications [1]. These phenolics, including phenolic acids, flavonoids, and proanthocyanidins, possess various health-promoting properties such as antioxidant, anti-inflammatory, and anticancer activities [2,3,4,5,6,7]. However, like many other plant matrices, grape pomace seeds contain a significant proportion of phenolic compounds existing in an insoluble form, especially after the maceration process of winemaking [8], which limits their further application based on the active phenolic compounds. Thus, there is a growing interest in developing strategies to enhance the release and bioavailability of phenolic compounds from grape seeds.

Soluble phenolics in plant matrices do not interact chemically or physically with other substances and can be easily extracted using polar water or organic solvents under mild conditions. However, a substantial proportion of insoluble phenolics in plant matrices is covalently bound to macromolecules in cell walls, such as cellulose, hemicellulose, lignin, pectin, and structural proteins, resulting in their insolubility and extremely low extractability in traditional organic solvents [9,10]. Through there are many means to extract insoluble phenolics, such as enzyme-assisted, microwave-assisted, and ultrasound-assisted hydrolysis approaches, chemical hydrolysis methods employing high concentrations of strong acid or strong alkali are the most commonly utilized methods [10,11,12]. However, these methods can lead to the destruction of the phenolic hydroxyl group structures of active phenolic compounds, thereby affecting their bioactivity. Moreover, the use of high concentrations of acids or bases can not only increase production costs but also pose environmental challenges, hindering their industrial application. SSF has emerged as a promising technique for modifying and improving the bioactive composition of diverse plant materials at a very low cost. Through the utilization of specific microorganisms, particularly fungi, SSF can facilitate the breakdown of the complex cell wall structure through the action of their abundant hydrolase enzymes, release soluble phenolics, and enhance the overall nutritional and functional properties of the fermented material [13,14,15]. In the context of grape seeds, SSF also presents a potential method for augmenting the release of soluble phenolics and enhancing antioxidant activity [16].

Therefore, the present study aimed to evaluate the impact of SSF with *Aspergillus niger* and two other fungi, namely *Monascus anka* and *Eurotium cristatum*, which are from Chinese traditional fermented foods, on the release of soluble phenolics from grape pomace seeds. In addition, the correlation between the phenolic compounds and hydrolase activities during SSF was assessed. The findings of this study are expected to provide valuable insights into the utilization of grape pomace seeds based on phenolic compounds in the food industry.

## 2. Materials and Methods

### 2.1. Sample Pretreatment

Grape pomace seeds were obtained from Cabernet Sauvignon pomace through manual sorting. After washing and drying, the grape pomace seeds were ground into powder and then sieved through an 18-mesh sieve. Before use, 500 mL of 80% methanol was added to 300 g grape pomace seed powder, and this mixture was incubated at 40 °C for 5 h to remove the soluble phenolics. The separation of the supernatant and iterative extraction of the powder residue was performed until the soluble phenolics were effectively removed. Then, the powder residues were dried at 50 °C for 12 h to yield grape pomace seed samples for SSF.

### 2.2. Bacterial Strains, Media, and Culture Conditions

*A. niger* CICC 2214 and *A. niger* CICC 41481 were all sourced from China Center of Industrial Culture Collection (CICC). *M. anka* GIM 3.592 was sourced from Guangdong Microbial Culture Collection Center (GDMCC). *Eurotium cristatum* FEc.1-1 was generously provided by Professor Ling Tiejun from Anhui Agricultural University. All four fungi were maintained on Potato Dextrose Agar (PDA) medium purchased from Solarbio (Shanghai, China). For solid-state fermentation (SSF), specific amounts of substrates were prepared for each strain. In Petri dishes with a diameter of 75 mm, the mixture of 0.5 g of rice flour and 3.0 g of grape pomace seed powder was used as the substrate for *M. anka* SSF, while the mixture of 0.8 g of rice flour and 3.0 g of grape pomace seed powder was used for *E. cristatum* SSF. Additionally, 1.0 g of grape pomace seed powder was used as the substrate for *A. niger* SSF.

### 2.3. SSF

Strain spores were washed from their PDA plates with 5 mL 0.1% Tween 80 solution. After filtering with lens papers, the spore suspensions were adjusted to a concentration of 3 × 10^6^ spores/mL. Then, 2.45 mL of the spore suspensions were incubated on SSF substrates for a 12-day fermentation at 28 °C and 65% humidity. The fermented samples were freeze-dried for 24 h, and then ground for subsequent analysis.

### 2.4. Extraction of Soluble and Insoluble Phenolics

To extract soluble phenolics, samples weighing 2.0 g were mixed with 40 mL of 80% methanol solution. The mixture was centrifuged at 10,000 rpm for 5 min after incubation in a 40 °C water bath for one hour. Subsequently, the residues were kept for extracting insoluble phenolics, and the supernatant was concentrated under vacuum by a rotary evaporator to a volume of 8 mL at 40 °C, followed by an adjustment of the volume to 10 mL with distilled water. The resulting solution was degreased using 20 mL of n-hexane and then extracted twice with 20 mL of ethyl acetate. The combined ethyl acetate extraction was evaporated under vacuum by a rotary evaporator to dryness at 40 °C. Then, the obtained residues were re-dissolved in 2 mL of methanol for further analysis. To extract insoluble phenolics, 40 mL of 4 M NaOH solution was added to the aforementioned residues. After incubation at room temperature for 4 h, the mixture underwent an adjustment of the pH to 2.0 with concentrated hydrochloric acid. Subsequently, the filtered mixture was extracted with filtration of 40 mL n-hexane and then extracted twice with 40 mL of ethyl acetate. The combined ethyl acetate extraction was evaporated under vacuum by a rotary evaporator to dryness at 40 °C. Then the obtained residues were re-dissolved in 2 mL of methanol for further analysis.

### 2.5. Determination of Total Phenolic and Total Flavonoid Content

The total phenolic content (TPC) was determined using the Folin–Ciocalteu method [16]. First, 0.5 mL of Folin–Ciocalteu reagent was mixed with 1 mL of the appropriately diluted extract, followed by the addition of 1.5 mL of 20% (*w*/*v*) Na_2_CO_3_ solution. After standing at room temperature for 5 min, the mixture volume was adjusted to 10 mL with deionized water and left at room temperature for one hour. The absorbance of the mixture was measured at 765 nm using a UV-1206 spectrophotometer (Shimadzu, Kyoto, Japan). Gallic acid (Aladdin Biochemical Technology Co., Ltd., Shanghai, China) was used to create the standard curve (Y = 0.0112X + 0.0522, R^2^ = 0.9986) for TPC analysis. The TPC was expressed as mg gallic acid equivalents (GAE) per 100 g sample dry weight (DW).

The total flavonoid content (TFC) was determined according to the method described by Lu Wang et al. [17]. Specifically, 200 µL of diluted extract was combined with 800 µL of 80% (*v*/*v*) ethanol solution and 60 µL of 5% (*w*/*v*) sodium nitrite solution. After standing at room temperature for 5 min, this mixture was supplied with 60 µL of 10% (*w*/*v*) aluminum chloride solution, followed by standing for 6 min. Subsequently, 400 µL of a 1.0 M sodium hydroxide solution and 480 µL of 80% (*v*/*v*) ethanol solution were added to react for 30 min at room temperature in the dark. The absorbance of the mixture was measured at 510 nm. A standard curve was generated using catechin (Aladdin Biochemical Technology Co., Ltd., China) (Y = 0.0027X − 0.0071, R^2^ = 0.999). The TFC was expressed as mg catechin equivalents (CE) per 100 g sample dry weight (DW).

### 2.6. Phenolic Composition Analysis

The phenolic composition of the samples was analyzed using a 1260 high-performance liquid chromatography (HPLC) system (Agilent, Santa Clara, CA, USA) with a diode array detector. A Sunfire C18 reverse phase chromatographic column (250 × 4.6 mm, 5 μm) (Waters, Milford, MA, USA) was used with a flow rate of 1 mL/min and a column temperature of 30 °C. The mobile phases A and B consisted of 2% (*v*/*v*) acetic acid solution (A) and 2% (*v*/*v*) acetic acid-acetonitrile solution, respectively. Gradient elution was used with the following conditions: 0 min, 5% B; 40 min, 20% B; 55 min, 27.5% B; 65 min, 50% B; 66–71 min, 80% B; 73–78 min, 5% B. The injection volume was set to 10 μL, and all phenolic compounds were detected at 200–650 nm. By comparing the retention times and UV spectra to the phenolic standards, including gallic acid, protocatechuic acid, procyanidins B1, catechin, chlorogenic acid, procyanidins B2, caffeic acid, syringic acid, epicatechin, ferulic acid, rutin, epicatechin gallate, resveratrol, and p-coumaric acid (Beijing Solarbio Science & Technology Co., Ltd., Beijing, China), the phenolic compounds in the samples were qualitatively identified. The content of individual phenolic was then calculated based on the peak area and standard curve [18].

### 2.7. DPPH and ABTS Radical Scavenging Capacities

To determine the DPPH radical scavenging capacity, 2 mL of a 100 µM DPPH^•^ solution was mixed with 250 µL of the diluted extractions of soluble and insoluble phenolics, and then the mixture was allowed to react in the dark for 30 min. The absorbance value of the mixture at 517 nm was measured. A standard curve (Y = −0.0025X + 0.9639, R^2^ = 0.9962) was generated using ascorbic acid (VC) (Xilong Scientific Co., Ltd., Shantou, China). The DPPH radical scavenging capacity was calculated as mg VC equivalents per 100 g of sample [19].

To determine the ABTS radical scavenging capacity, 2.45 mM potassium persulfate solution and 7 mM ABTS^•+^ were mixed evenly in equal volume, and this mixture was allowed to react at room temperature for 16 h without light. The mixture was then diluted with ethanol until reaching an absorbance of 0.70 ± 0.02 at 734 nm to prepare the ABTS^•+^ solution. Subsequently, 2 mL of the ABTS^•+^ solution was added to 500 μL of the diluted extractions of soluble and insoluble phenolics, and the mixture was allowed to react at room temperature in the dark for 10 min. A standard curve was also created using VC (Y = −0.0050X + 0.5724, R^2^ = 0.9994). The absorbance of the mixture was then measured at 734 nm. The ABTS radical scavenging capacity was also calculated as mg VC equivalents per 100 g of sample [19].

### 2.8. Extraction of Crude Enzyme from Solid-State Fermentation Samples

To extract crude enzyme from the samples, 20 mL of sodium citrate buffer (50 mM, pH 5.5) was introduced. The resulting mixture was then shaken at 150 rpm and 30 °C for 30 min. Subsequently, the crude extract was centrifuged at 4 °C for 20 min at a speed of 10,000 rpm, and the resulting supernatant was collected for further enzyme activity analysis.

### 2.9. Determination of Hydrolase Activities during SSF

To prepare the DNS solution, initially, 185.0 g of potassium sodium tartrate was dissolved in 500 mL of distilled water at 50 °C. Subsequently, 6.3 g of 3,5-dinitrosalicylic acid, 21.0 g of sodium hydroxide, 5.0 g of phenol, and 5.0 g of sodium sulfite were sequentially added to the solution. After ultrasonic dissolution in the dark, the volume was adjusted to 1 L with distilled water.

To investigate xylanase activity, 500 μL of the crude enzyme solution was combined with 500 mL of a 1% xylan solution (*w*/*v*) prepared using 50 mM citric acid buffer (pH 4.8) and mixed thoroughly. After incubation in a 50 °C constant water bath for 30 min, 100 μL of the reaction solution was collected and mixed with 900 μL of distilled water and 3.0 mL of DNS solution. The mixture was boiled for 5 min, cooled to room temperature, added with 20 mL distilled water, and thoroughly mixed. The absorbance of the solution at 540 nm was then measured.

To evaluate pectinase activity, 100 μL of the crude enzyme solution was mixed with 0.5 mL of polygalacturonic acid solution (1%, *w*/*v*) prepared in citric acid buffer (pH 4.8) and 1.4 mL of citric acid buffer (pH 4.8). The mixture was incubated in a water bath at 37 °C for 20 min, following which 3 mL of DNS solution and 1 mL of distilled water were added. Absorbance was measured at 575 nm after the solution cooling.

To assess cellulase activity, 50 mg of filter paper was weighed and mixed with 0.5 mL of the diluted crude enzyme solution and 1 mL of sodium citrate buffer solution (50 mM, pH 4.8), and incubated in a water bath for 30 min at 50 °C. The reaction was terminated by adding 3.0 mL of DNS solution to the mixture, followed by boiling for 5 min in a water bath. After adding 20 mL of distilled water to the mixture, the absorbance was measured at 540 nm.

To measure *β*-glucosidase activity, 200 μL of the diluted crude enzyme solution, along with 100 μL of p-nitrophenyl-D-glucopyranoside (PNPG) solution (9 mM) and 800 μL of sodium acetate buffer (200 mM, pH 4.8), were mixed thoroughly. The resulting mixture was incubated in a 45 °C water bath for 30 min, following which 500 μL of 1 M Na_2_CO_3_ was added to terminate the reaction. The absorbance of the solution was measured at 420 nm.

The activity of all enzymes is defined as the quantity of enzyme required to release 1 μmol of product per minute per gram of fermentation substrate under experimental conditions, and is expressed in units per gram (U/g).

### 2.10. Statistical Analysis

All test data are expressed as the mean ± standard deviation (SD) of three independent experiments. Significant differences were analyzed by one-way analysis of variance (ANOVA) or by Student’s *t*-tests. A level of *p* < 0.05 was considered statistically significance in all cases.

## 3. Results and Discussion

### 3.1. Enchaned Release of Soluble Phenolics of Grape Pomace Seeds via SSF with Different Fungi

#### 3.1.1. TPC and TFC

**Figure 1:**
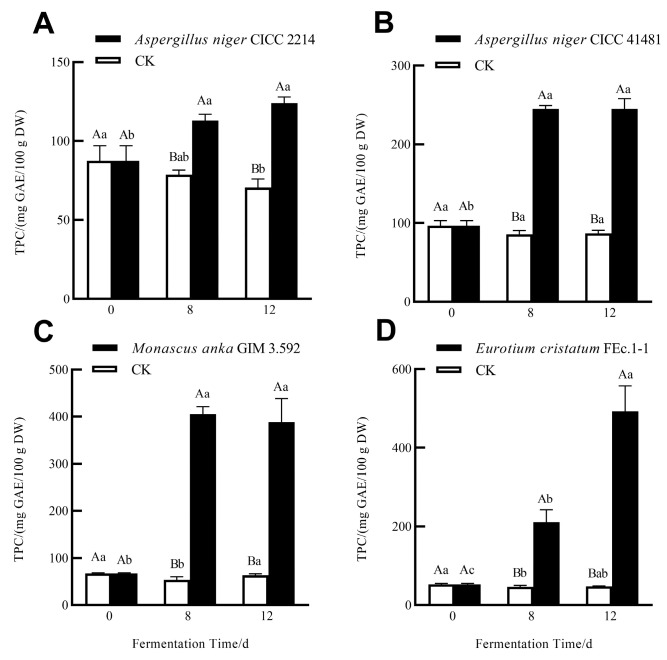
Total phenolic content (TPC) of soluble phenolics of grape pomace seeds without SSF (CK) and during SSF with *A. niger* CICC 2214 (**A**), *A. niger* CICC 41481 (**B**), *M. anka* GIM 3.592 (**C**), and *E. cristatum* FEc.1-1 (**D**). Different capital letters represent significant differences (*p* < 0.05) between the samples from the same fermentation time. Different lower-case letters represent significant differences (*p* < 0.05) among the samples from the same treatment.

**Figure 2:**
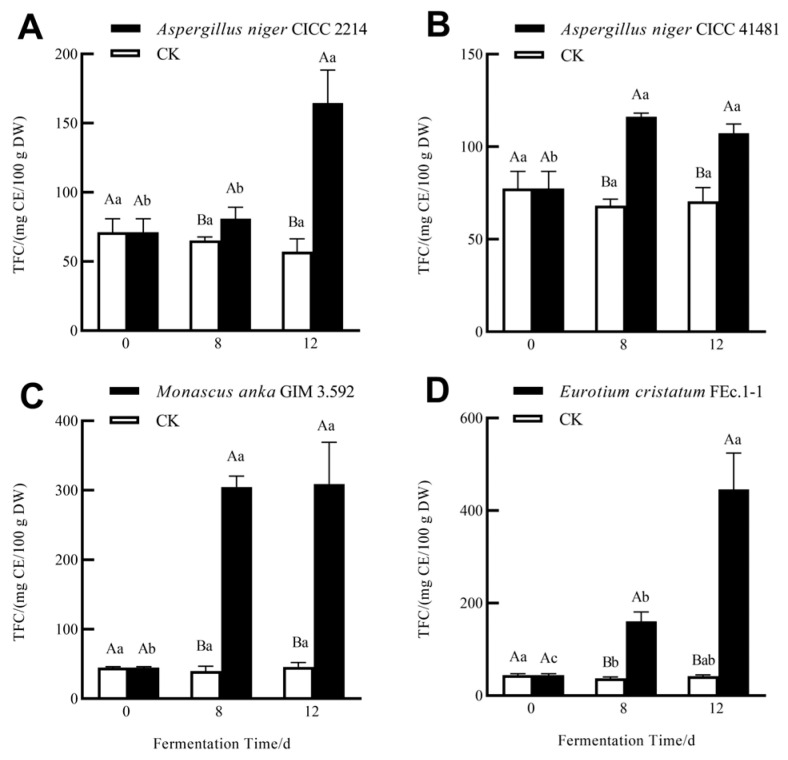
Total flavonoid content (TFC) of soluble phenolics of grape pomace seeds without SSF (CK) and during SSF with *A. niger* CICC 2214 (**A**), *A. niger* CICC 41481 (**B**), *M. anka* GIM 3.592 (**C**), and *E. cristatum* FEc.1-1 (**D**). Different capital letters represent significant differences (*p* < 0.05) between the samples from the same fermentation time. Different lower-case letters represent significant differences (*p* < 0.05) among the samples from the same treatment.

The effects of SSF with different fungi on TPC and TFC of soluble phenolics in grape pomace seeds are shown in Figure 1. After 8-day and 12-day SSF, TPC and TFC of the soluble phenolic fraction significantly increased. Among the four tested fungi, *E. cristatum* FEc.1-1 exhibited the highest ability to release soluble phenolics, with TPC increasing by 9.21 times and TFC increasing by 10.08 times after 12-day SSF (Figure 1D and Figure 2D). *M. anka* GIM 3.592 followed, with TPC increasing by 5.72 times and TFC increasing by 6.88 times (Figure 1C and Figure 2C). Compared to *E. cristatum* and *M. anka*, two *A. niger* strains released relatively few soluble phenolics (Figure 1). Two *A. niger* strains also showed a different effect on TPC and TFC of the soluble fraction, with TPC increasing by 2.53 times (CICC41481) and 1.41 times (CICC2214), and TFC increasing by 2.31 times (CICC41481) and 1.39 times (CICC2214). This indicated that the ability of fungi to release phenolics via SSF was strain-dependent. Similar results were obtained with *A. niger* NRRL3 and *A. niger* 3T5B8 during the grape pomace SSF process, with differences in the release of soluble phenolics and antioxidant capacity [16,20].

In terms of TPC and TFC, 12-day SSF with *A. niger* CICC41481 and *M. anka* GIM 3.592 showed no difference compared to 8-day SSF. However, 12-day SSF with *A. niger* CICC2214 and *E. cristatum* showed higher levels than their 8-day SSF. This indicates that the fermentation duration of *A. niger* CICC41481 and *M. anka* GIM 3.592 for soluble phenolics was shorter than that of *A. niger* CICC2214 and *E. cristatum*. Many studies have demonstrated that different hydrolase enzymes produced by fungi can be responsible for the increased soluble phenolics and antioxidant activities by fungal SSF [15,16]. More direct evidence for the roles of hydrolase enzymes from the literature shows that enzyme-assisted extraction for phenolics in grape pomaces, instead of solvent extraction, can yield higher concentrations of soluble phenolics and higher biological activities [20,21]. These improvements caused by fungal SSF can be attributed to the direct action of hydrolase enzymes on the cell wall compositions of plant matrices to release the bound phenolics.

#### 3.1.2. Individual Phenolics

Numerous previous studies have demonstrated the close association between the antioxidant potential of plant substrates and the abundance of diverse phenolic compounds [22,23,24,25]. In this study, HPLC was employed to analyze 13 individual phenolics present in soluble form in grape pomace seeds (Table 1). The results indicate that SSF with fungi significantly impacts the composition and content of soluble phenolics in grape pomace seeds. Specifically, after SSF with fungi, the concentrations of several individual phenolics in grape pomaces seeds, such as gallic acid and protocatechuic acid, significantly decreased, consistent with *Psidium guajava* L. leaf SSF, as previously reported [26]. It has been reported that microorganisms can metabolize aromatic compounds by utilizing them as carbon sources through the ring cleavage pathway [27]. Gallic acid and protocatechuic acid can also serve as center ring fission intermediates and enter the tricarboxylic acid (TCA) cycle via the aromatic metabolic pathway [28].

Among the four strains studied, SSF with *M. anka* resulted in significant increases in the majority of individual phenolics in grape pomace seeds, including proanthocyanidin B1, catechin, chlorogenic acid, syringic acid, ferulic acid, epicatechin gallate, and resveratrol. These compounds have demonstrated beneficial effects, such as anti-cardiovascular, neuroprotective, and anti-inflammatory effects [2,3,4,5,29]. In particular, for chlorogenic acid, SSF with *M. anka* increased its content by about 400 times. As for ferulic acid, it can be said that SSF with *M. anka* increased its content from zero to a higher level of 6.15 mg/Kg, compared to SSF with other fungi. This was followed by *E. cristatum*; its SSF significantly increased the contents of procyanidins B1, chlorogenic acid, syringic acid, ferulic acid, rutin, and epicatechin gallate. The two *A. niger* strains showed poor performance in releasing individual phenolics, with significant decreases in the contents of most phenolics after SSF. However, it is notable that the content of caffeic acid was found to increase after SSF with *A. niger*, while, in contrast, it was not detected before and after SSF with *M. anka* or *E. cristatum*. A ferulic acid metabolism pathway exists in the bacteria *Enterobacter cloacae* and *Streptomyces albogriseolus*, the ascomycete *Penicillium rubens*, and the yeast *Saccharomyces cerevisiae*, and previous studies have shown that ferulic acid can be demethylated to caffeic acid or p-coumaric acid, etc. [28]. Based on this, it is speculated that *A. niger* may possess a robust ferulic acid metabolic enzyme system to form more caffeic acid, compared to *M. anka* and *E. cristatum*.

### 3.2. Enhanced Antioxidant Activity of Soluble Phenolics of Grape Pomace Seeds via SSF with Different Fungi

**Figure 3:**
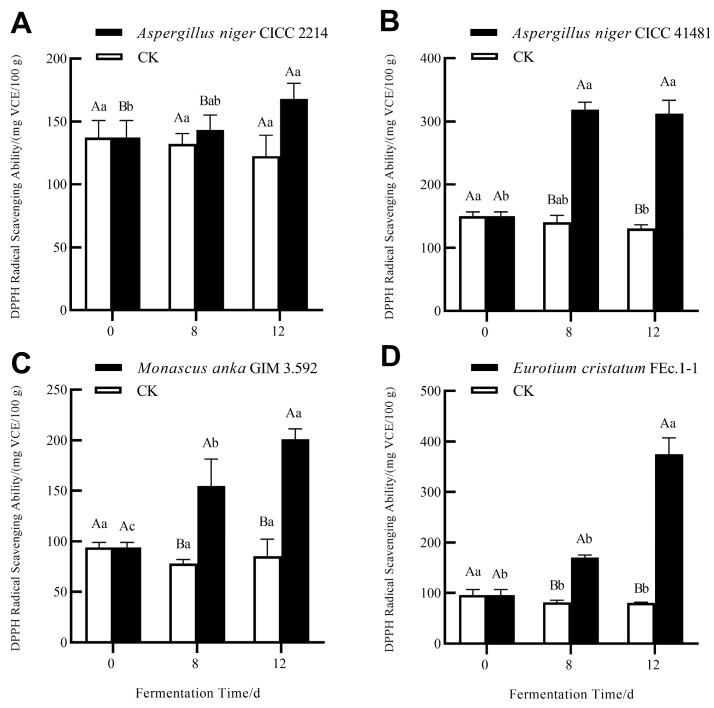
DPPH radical scavenging activities of soluble phenolics of grape pomace seeds during SSF with *A. niger* CICC 2214 (**A**), *A. niger* CICC 41481 (**B**), *M. anka* GIM 3.592 (**C**), and *E. cristatum* FEc.1-1 (**D**). Different capital letters represent significant differences (*p* < 0.05) between the samples from the same fermentation time. Different lower-case letters represent significant differences (*p* < 0.05) among the samples from the same treatment.

**Figure 4:**
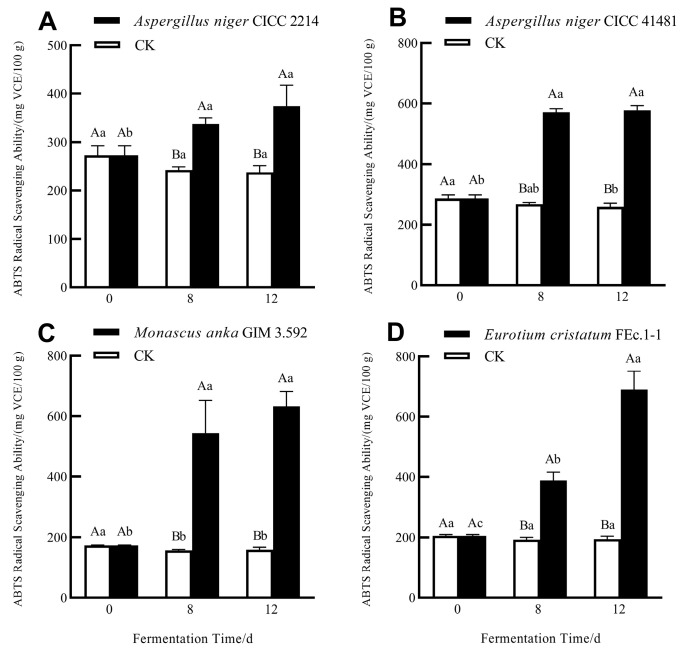
ABTS radical scavenging activities of soluble phenolics of grape pomace seeds during SSF with *A. niger* CICC 2214 (**A**), *A. niger* CICC 41481 (**B**), *M. anka* GIM 3.592 (**C**), and *E. cristatum* FEc.1-1 (**D**). Different capital letters represent significant differences (*p* < 0.05) between the samples from the same fermentation time. Different lower-case letters represent significant differences (*p* < 0.05) among the samples from the same treatment.

In this study, DPPH and ABTS radical scavenging capacities were employed to assess the antioxidant activities of the samples. As demonstrated in Figure 3 and Figure 4, SSF with four strains significantly enhanced the DPPH and ABTS radical scavenging potential of soluble phenolics in grape pomace seeds. This enhancement has also been found in the SSF of grape pomaces using *Zygomycetes* [30]. A 12-day SSF with *E. cristatum* resulted in the highest improvement of the DPPH radical scavenging activity, with a significant increase of 3.91-fold. However, a 12-day SSF with *M. anka* gave the highest improvement of the ABTS radical scavenging capacity, with a significant increase of 3.64-fold. As highlighted in a previous study [31], there is a direct correlation between antioxidant activity and phenolic content of plant matrix extract. In the present study, antioxidant activity changes in fermented samples showed the same increasing trend of TPC and TFC. However, for the samples fermented by different fungi, the correlation between antioxidant activity and TPC, TFC, or individual phenolic content is not strong. This might be related to the fact that the antioxidant capacity of phenolic compounds depends not only on their content, but also on their donor proton capacity and aromatic ring electron delocalization ability [32]. In addition, the existence of many complex phenolics in grape seeds, such as varieties of oligomeric and polymeric proanthocyanidins (syn. condensed tannins) that cannot be easily quantified, may also impact the antioxidant capacity of the phenolic extracts [33].

### 3.3. Dynamic Changes in Soluble Phenolics, Insoluble Phenolics, and Antioxidant Activities of Grape Pomace Seeds during SSF with M. anka

**Figure 5:**
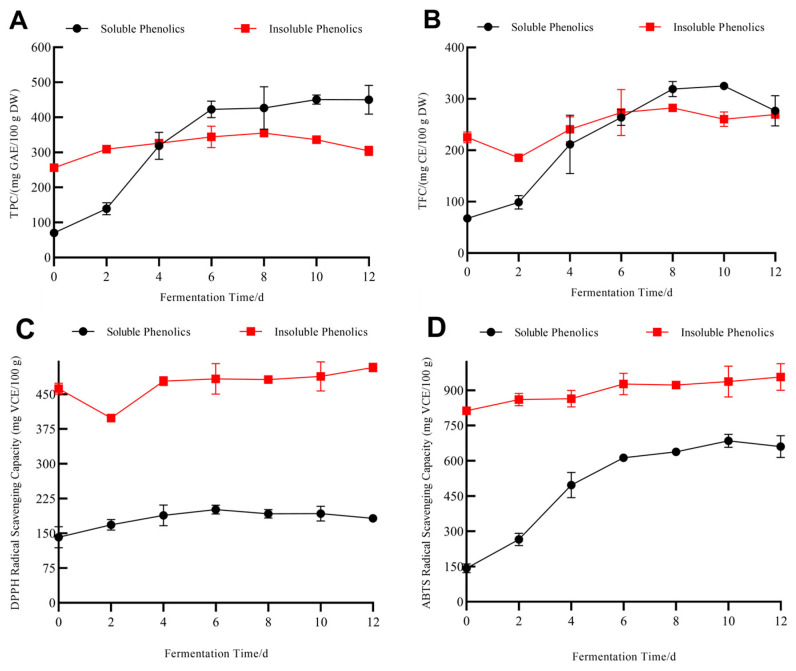
Dynamic changes in TPC (**A**), TFC (**B**), DPPH radical scavenging capacity (**C**), and ABTS radical scavenging capacity (**D**) of soluble and insoluble phenolics of grape pomace seeds during SSF with *M. anka*.

Similar to previous reports [15], SSF with the four fungi tested in this study can release soluble phenolics and enhance the antioxidant activities in grape pomace seeds. In this section, insoluble phenolic changes in grape pomace seeds during SSF with *M. anka* were also investigated. The changes in soluble phenolics and the corresponding antioxidant activities (Figure 5) were consistent with the results in Section 3.1. The content and antioxidant activities of soluble phenolics increased significantly during SSF. After 10-day SSF with *M. anka*, the TPC and TFC of soluble phenolics increased from 70.00 to 450.01 mg GAE/100 g DW (Figure 5A,B), and from 67.58 to 325.20 mg CE/100 g DW.

Due to the removal of soluble phenolics in the pretreatment of grape pomace seeds, before SSF, the TPC and TFC of the insoluble phenolics in grape pomace seeds were significantly higher than those of the soluble phenolics (Figure 5A,B). However, this situation completely reversed after SSF due to the enhanced release of the soluble phenolics by *M. anka*. Unlike the huge changes in the soluble phenolics during SSF, the insoluble phenolics just showed a slight increase. This indicates the soluble phenolics in grape pomace seed are more susceptible to fungal SSF than the insoluble ones, which was also demonstrated in the SSF of oats (*Avena sativa* L.) with *M. anka* [17].

### 3.4. Dynamic Changes in Hydrolase Activities during SSF with M. anka

**Figure 6:**
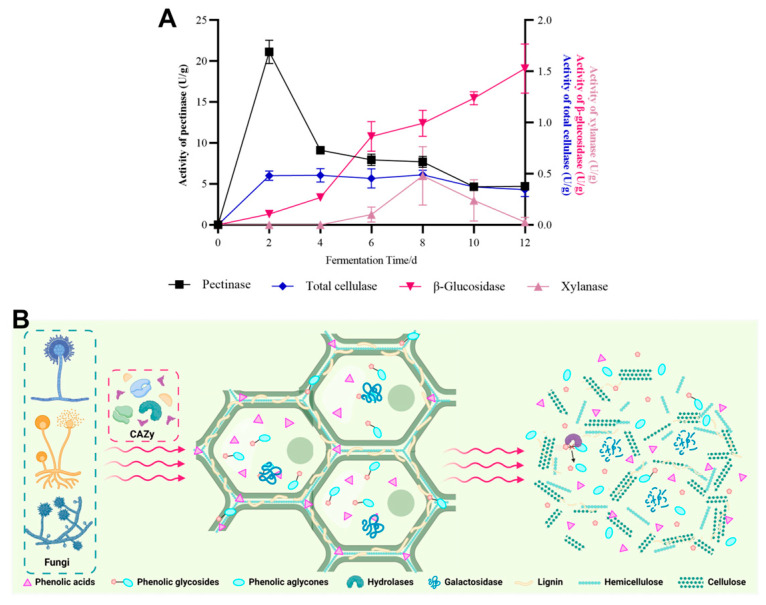
Changes in hydrolase activities during SSF of grape pomace seeds with *M. anka* (**A**); hypothetical mechanism of hydrolase action on soluble phenolic release (**B**).

During SSF, the activities of some hydrolases from the microorganisms have been confirmed to be directly associated with the release of soluble phenolics in plant matrices in numerous studies [17,27,34]. Additionally, direct hydrolase enzyme treatments also enhance the release of soluble phenolics from plant substrates and their antioxidant activities [9,35,36,37]. This enhancement can be attributed to the mechanism in which the hydrolases weaken the chemical bond between insoluble phenolics and cell wall elements, making it easier to extract these insoluble compounds, and then leading to an increase in the content and antioxidant activity of soluble phenolics (Figure 6B).

During SSF with *M. anka*, the activities of four hydrolases reported to be related to the release of soluble phenolics were investigated. As depicted in Figure 6A, during SSF, the activities of these hydrolases exhibited dynamic changes throughout the 12-day SSF period. Notably, *β*-glucosidase activity exhibited a constant increase throughout the entire SSF period, contrasting with the declining trends observed for the activities of three other hydrolases (pectinase, cellulase, and xylanase) after reaching their respective peak values during SSF. This trend may be attributed to a consequence of diminished nutrient availability for microorganisms, resulting in reduced hydrolase production during SSF [38]. *β*-glucosidase has been reported to be beneficial for the hydrolysis of many phenolic and flavonoid glycosides, such as isoquercetin (quercetin-3-O-glucoside), polydatin (resveratrol-3-O-glucoside), and astragalin (kaempferol-3-O-glucoside), resulting in the release of aglycones with high biological activity in grape pomace seeds [39]. In this study, there was indeed a strong positive correlation between *β*-glucosidase activity and soluble phenolic content (r = 0.900) during SSF. Notably, the significant increase in resveratrol content after SSF (Table 1) further supports this correlation. Previous reports also suggest that SSF with filamentous fungi, such as *M. anka*, *Aspergillus oryzae*, and *Aspergillus sojae*, leads to heightened extraction rates of phenolics from plant matrices due to the high *β*-glucosidases activities of these fungi [15,16]. The pectinase activity reached its highest level (21.13 U/g) on the second day of SSF. In the present study, during SSF with *M. anka*, pectinase activity showed a low negative correlation (r = −0.203) to TPC of soluble phenolic content. The correlations between pectinase activity and phenolic content have been reported to be different in SSF with different fungi. For instance, in the SSF of grape pomaces with *A. niger* and *Aspergillus oryzae*, a contrasting pattern was observed, with a strong positive correlation for *A. niger* and a strong negative correlation for *A. oryzae* [16]. Compared to the high cellulase activities reported for *A. niger* and *A. oryzae* during SSF [16], those of *M. anka* during SFF maintained low values with a maximum of 0.49 U/g. Additionally, the xylanase activity was only detected during the 6–10 days of SSF, with a maximum of only 0.48 U/g at day 8. The two hydrolase activities showed moderate positive correlations to phenolic contents, with r = 0.514 for cellulase and r = 0.558 for xylanase. These findings indicate a close relationship between the increased contents of soluble phenolics during SSF and the hydrolases produced by microorganisms.

## 4. Conclusions

In summary, SSF with different fungi can increase the soluble phenolic contents of grape pomace seeds, thereby improving their antioxidant abilities to varying degrees. In particular, SSF with *M. anka* GIM 3.592 and *E. cristatum* FEc.1-1 showed the strongest ability to release soluble phenolics in grape pomace seeds. Additionally, SSF with *M. anka* GIM 3.592 exhibited the greatest enhancement in the contents of individual phenolics, especially for chlorogenic acid, syringic acid, ferulic acid, epicatechin gallate, and resveratrol. Furthermore, our investigation revealed direct positive correlations between hydrolase activities and soluble phenolic contents. These correlations underline the influence of enzymatic activities, particularly those of *β*-glucosidase, on the liberation of phenolic compounds in grape pomace seeds. In conclusion, our findings highlight the potential of SSF with *M. anka* to effectively improve the phenolic-based quality of grape pomace seeds, showcasing the promising applications in improving the nutritional and functional attributes of this by-product.

## Figures and Tables

**Table 1:** Individual phenolic contents of grape pomace seeds without SSF (CK) and after 12-day SSF with different fungi.

Individual Phenolics	*A. niger* CICC 2214 CK	*A. niger* CICC 2214	*A. niger* CICC 41481 CK	*A. niger* CICC 41481	*M. anka* GIM3.592 CK	*M. anka* GIM3.592	*E. cristatum* FEc.1-1 CK	*E. cristatum* FEc.1-1
Gallic acid	27.25 ± 0.70 A	ND	37.36 ± 5.25 A	ND	17.77 ± 0.36 A	ND	15.28 ± 1.03 A	ND
Protocatechuic acid	3.19 ± 0.03 A	ND	4.91 ± 0.62 A	ND	1.43 ± 0.03 A	0.45 ± 0.09 B	1.46 ± 0.23 A	ND
Procyanidins B1	0.70 ± 0.21 A	ND	1.24 ± 0.37 A	0.71 ± 0.26 B	0.90 ± 0.03 B	1.17 ± 0.07 A	0.58 ± 0.08 B	1.20 ± 0.37 A
Catechin	18.00 ± 0.17 A	ND	16.67 ± 3.51 A	ND	14.09 ± 0.29 B	34.52 ± 4.12 A	10.86 ± 0.75 A	1.91 ± 0.16 B
Chlorogenic acid	ND	2.20 ± 0.18 A	ND	11.12 ± 2.20 A	0.18 ± 0.00 B	80.56 ± 1.73 A	ND	0.05 ± 0.00 A
Procyanidins B2	2.92 ± 0.19 A	ND	3.88 ± 0.07 A	ND	2.31 ± 0.00 A	1.55 ± 0.5 A	1.63 ± 0.20 A	1.47 ± 0.10 A
Caffeic acid	ND	2.47 ± 0.42 A	ND	2.15 ± 0.26 A	ND	ND	ND	ND
Syringic acid	0.67 ± 0.06 A	ND	0.78 ± 0.17 A	ND	0.35 ± 0.01 B	2.85 ± 0.09 A	0.53 ± 0.08 B	3.36 ± 0.16 A
Epicatechin	9.55 ± 0.36 B	*	9.06 ± 1.18 B	*	8.36 ± 0.78 A	9.94 ± 0.48 A	6.09 ± 0.60 A	1.66 ± 0.16 B
Ferulic acid	ND	0.74 ± 0.05 A	ND	0.95 ± 0.04 A	ND	6.15 ± 0.09 A	ND	0.90 ± 0.15 A
Rutin	4.61 ± 0.19 A	ND	6.65 ± 1.34 A	1.09 ± 0.02 B	5.07 ± 1.19 A	0.27 ± 0.03 B	3.48 ± 0.85 B	5.73 ± 0.09 A
Epicatechin gallate	ND	ND	ND	ND	ND	2.59 ± 0.30 A	ND	3.68 ± 0.73 A
Resveratrol	0.01 ± 0.00 A	0.08 ± 0.06 A	0.09 ± 0.02 B	0.30 ± 0.08 A	0.01 ± 0.00 B	0.14 ± 0.03 A	0.03 ± 0.00 B	0.11 ± 0.00 A

“ND” indicates that it is below the detection limit and not detected. Different capital letters within a row indicate a significant difference between the CK and treatment (*p* < 0.05). “*” represents that there are other substances derived from fungal metabolism with high response at the peak position of this substance.

## Data Availability

The original contributions presented in the study are included in the article, further inquiries can be directed to the corresponding author.
